# The Predictive Value of Pan-Immune-Inflammation Value for Saphenous Vein Graft Disease in Post-Coronary Artery Bypass Grafting Patients

**DOI:** 10.3390/jcdd11110337

**Published:** 2024-10-22

**Authors:** Faruk Serhatlioglu, Zeki Cetinkaya, Yucel Yilmaz

**Affiliations:** 1 Department of Cardiovascular Surgery, Faculty of Medicine, Nigde Omer Halisdemir University, Nigde 51240, Turkey; faruks@erciyes.edu.tr; 2 Department of Cardiology, Ministry of Health, Elazıg Fethi Sekin City Hospital, Elazıg 23280, Turkey; zeki2387@gmail.com; 3 Department of Cardiology, Kayseri City Training and Research Hospital, University of Health Sciences, Kayseri 38080, Turkey

**Keywords:** saphenous vein graft stenosis, pan-immune-inflammation value, inflammation

## Abstract

**Background:** Coronary artery bypass grafting (CABG) remains the gold standard treatment for patients with significant coronary artery disease (CAD) and high-risk profiles for percutaneous coronary intervention. Despite the frequent use of saphenous vein grafts (SVGs) in CABG, their patency rates are lower than those of arterial grafts. Identifying noninvasive methods to determine SVG patency is crucial. **Aims:** This study investigates the relationship between a novel inflammation marker, pan-immune-inflammation value (PIV), and SVG patency in post-CABG patients. **Methods:** The study included 507 patients who underwent coronary angiography (CAG) due to clinical indications between 2016 and 2023. Patients who had undergone CABG at least one year prior with at least one SGV used were divided into two groups based on the presence or absence of SVG stenosis (SGVS). **Results:** Among the 507 patients, 244 had SVGS. Patients with SVGS exhibited higher levels of diabetes mellitus and inflammatory markers such as NLR, SII, CAR, and PIV. Multivariate analysis identified PIV as an independent predictor of SVGS. ROC analysis showed that a PIV cut-off value > 315.5 predicted SVGS with 75.8% sensitivity and 68.6% specificity. **Conclusions:** PIV, a simple and easily measurable marker, demonstrated strong predictive value for SVGS in post-CABG patients.

## 1. Introduction

According to guidelines from the the American College of Cardiology (ACC)/American Heart Association (AHA) and the European Society of Cardiology (ESC), patients with coronary artery disease (CAD) who have a significant coronary plaque burden and/or are considered high risk for percutaneous coronary intervention (PCI) (such as those with complex multivessel coronary artery disease and/or left main coronary artery disease, patients with diabetes, or those with reduced left ventricular function) continue to be treated with coronary artery bypass grafting (CABG) surgery as the gold standard [1,2].

In this operation, both arterial and venous grafts can be used; however, it has been observed that the patency rates of saphenous vein grafts (SVGs) are significantly lower compared to those of the internal mammary artery (IMA). Although the patency rates for these vessels are 80–90% at 1 year and approximately 50% at 10 years, SVGs are still widely used due to their easy accessibility [2,3,4]. Therefore, the identification of noninvasive methods to determine SVG patency is of significant importance.

Inflammation plays a critical role at all stages of atherosclerosis/atherosclerotic diseases. Studies have shown that increased levels of inflammation markers provide information about the severity and prognosis of CAD. Moreover, hematological indices such as the neutrophil–lymphocyte ratio (NLR), monocyte–lymphocyte ratio (MLR), and systemic immune-inflammation index (SII) have been shown to be significant predictors of all-cause mortality following off-pump coronary artery bypass grafting (OPCABG) and for determining SVG patency after CABG [5,6,7,8].

The pan-immune-inflammation value (PIV), a new inflammation parameter recently introduced, has aroused curiosity regarding its potential to indicate the level of inflammation and its relationship to disease in patients with CAD. In our study, we aimed to investigate the relationship between PIV and SVG patency in patients undergoing CABG surgery.

## 2. Material and Methods

The study included 507 CABG patients who were discharged from the cardiology department of our hospital following coronary angiography (CAG) between 1 January 2016, and 12 July 2023. The patients were those who underwent CAG due to clinical indications of typical chest pain and/or results of noninvasive stress tests suggestive of myocardial ischemia (positive stress test results and/or ischemia on myocardial perfusion scintigraphy).

Patients with chronic coronary syndrome (CCS) who were diagnosed according to the 2019 ESC CCS diagnosis and treatment guidelines and who had undergone CABG surgery at least one year ago using at least one saphenous graft were included in this study [9]. SVG stenosis (SVGS) was defined as narrowing of 50% or more, and patients were divided into two groups: those with stenosis and those without stenosis.

Exclusion criteria included those presenting with a diagnosis of acute coronary syndrome or a history of acute coronary syndrome within the last three months and patients with left IMA disease. Additionally, patients with decompensated heart failure, severe heart valve disease, hematological disease, malignancy, severe pulmonary disease, severe renal (estimated glomerular filtration rate [eGFR] < 30 mL/min/1.73 m^2^) or liver disease, ongoing infections, or chronic inflammatory and autoimmune diseases were not included in the study.

The Research Ethics Institute of our hospital reviewed this study involving human subjects. The study was conducted in accordance with the guidelines set forth in the Declaration of Helsinki. The ethical committee was informed of the non-experimental design of the retrospective investigation and endorsed the study. An informed consent waiver was approved by the ethical committee due to the retrospective design of this study based on patient records.

### 2.1. Evaluation of the Coronary Angiography

**Figure 1:**
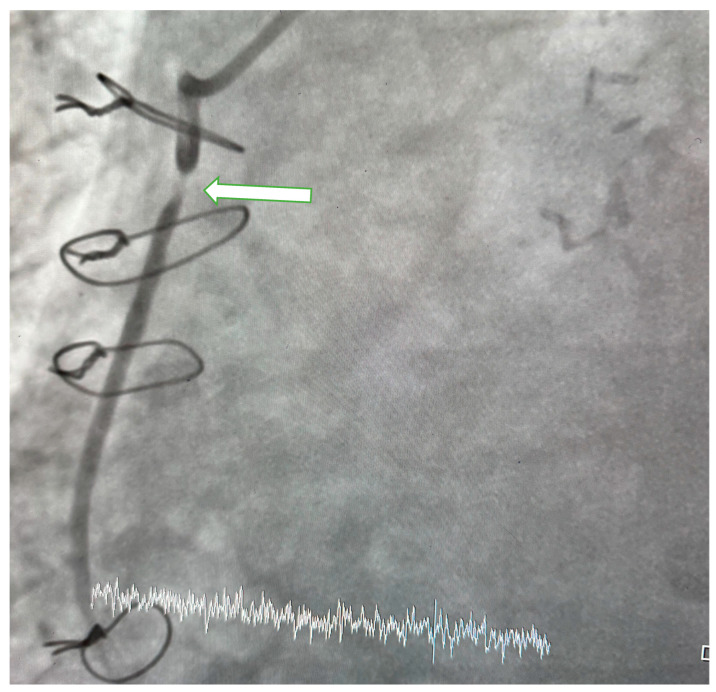
Coronary angiogram. In the image obtained at LAO36/CRA 0 degree of aorta-RCA saphenous vein graft (SVG), 95–99% stenosis is observed in the proximal segment of RCA-SGV (arrow).

Patients underwent CAG performed by cardiologists with physician or higher academic titles and who were unaware of the clinical details of the patients. CAG was carried out through radial or femoral artery approach to assess the degree of stenosis in the coronary arteries, their main branches, and graft vessels. When indicated, an evaluation of the aortic root was also performed. SVGS was defined as narrowing of 50% or more (Figure 1).

### 2.2. Laboratory Analysis

In all patients, blood samples were collected between 08:00 and 10:00 A.M. following a 12 h fasting period prior to the CAG. Antecubital venous blood samples were drawn into tubes containing tripotassium EDTA as an anticoagulant. Venous blood samples measuring basic blood variables (such as comprehensive metabolic panel and complete blood count) and thiol levels were obtained. All routine biochemical tests were performed on an autoanalyzer (Roche Diagnostic Modular Systems, Tokyo, Japan). Hematological parameters were stored at 4 °C and evaluated within 30 min of sampling using a Sysmex K-1000 autoanalyzer. NLR was calculated by dividing the number of neutrophils by the number of lymphocytes. SII was determined by multiplying the platelet count by NLR. PIV was calculated as the product of neutrophil count, platelet count, and monocyte count, divided by lymphocyte count (or by multiplying the monocyte count with SII).

### 2.3. Transthoracic Echocardiography

Transthoracic echocardiography was performed on each patient prior to coronary angiography. All measurements were conducted using a device equipped with a 3.5 MHz transducer (Vivid 5, GE Medical System, Horten, Norway) available at our hospital. Two-dimensional echocardiographic measurements were obtained to assess left ventricular ejection fraction and valve pathologies. The Simpson method was used to evaluate the ejection fraction from the apical four-chamber view, and color Doppler echocardiography was employed to assess valve pathologies.

### 2.4. Statistical Analyses

Statistical analyses were performed using SPSS 21.0 for Windows (SPSS Inc., Chicago, IL, USA). The distribution of quantitative variables was checked with the Shapiro–Wilk test. Descriptive data, depending on the normality of the distribution, were presented as mean ± standard deviation and median (interquartile range, IQR). For variables not following a normal distribution, median and interquartile ranges were provided. The independent samples *t*-test was used for comparing normally distributed quantitative variables, and the Mann–Whitney U test was used for non-normally distributed quantitative variables. Categorical variables were compared using the chi-square test. The effects of different variables on the development of SVGS were calculated with univariate analysis. For multivariate regression analysis, parameters with a *p*-value < 0.10 in univariate analysis were included in the model. However, to avoid multicollinearity, parameters that interact with each other were not entered into the model (e.g., platelet count, SII, NLR, and PIV), and thus, multivariate regression analysis was carried out separately with inflammatory parameters. The cut-off levels for PIV, SII, and C-reactive protein/albumin ratio (CAR) in predicting disease occurrence were determined by receiver operating characteristic (ROC) curve analysis. *P*-values below 0.05 were considered to indicate statistical significance.

G-power was used to determine the sample size. A minimum of 176 participants are needed for our study, with an alpha error of 0.05 and a statistical power of 0.80. A total of 542 patients (341 males) were included in the study. Of these, 263 had patent SGV, while 244 exhibited SVGS.

## 3. Results

The patients’ initial clinical and demographic parameters are detailed in Table 1. A total of 507 patients, with 341 being males, were enrolled in the study. Among all participants, 263 had patent SVG, while 244 exhibited SVGS. There was no difference between the two groups in terms of the number of SVG (2.14 ± 0.61 vs. 2.06 ± 0.71 *p* = 0.451) and LIMA numbers for anastomosis (242 (99.1%) 261 (99.2%) *p* = 0.882). The SVGS group had a higher prevalence of diabetes mellitus (DM) (*p* = 0.025), whereas other variables were comparable between the two groups. Laboratory variables are presented in Table 2. Patients with SVGS demonstrated higher platelet counts and lower albumin levels (*p* < 0.001, *p* = 0.012, respectively). Assessment of inflammation markers revealed elevated levels of NLR, CAR, and SII in patients with SVGS (*p* < 0.001 for all). The levels of PIV, as evaluated in this study, were statistically significantly higher in the SVGS patient group (*p* < 0.001). When evaluating angiographic data, it was observed that the time elapsed since CABG surgery was longer in patients with SVGS; aside from that, the two groups were comparable (*p* < 0.001) (Table 3).

The effectiveness of risk factors for SVGS was assessed through multivariate analysis. Multivariate logistic regression analysis was conducted using variables that demonstrated an association with disease formation in the univariate analysis, including DM, platelet count, NLR, albumin, CAR, SII, PIV, and the average time after CABG surgery. In the multivariate logistic regression analysis, SII, NLR, CAR, the average time after CABG surgery, and PIV were identified as independent determinants of SVGS (Table 4).

**Figure 2:**
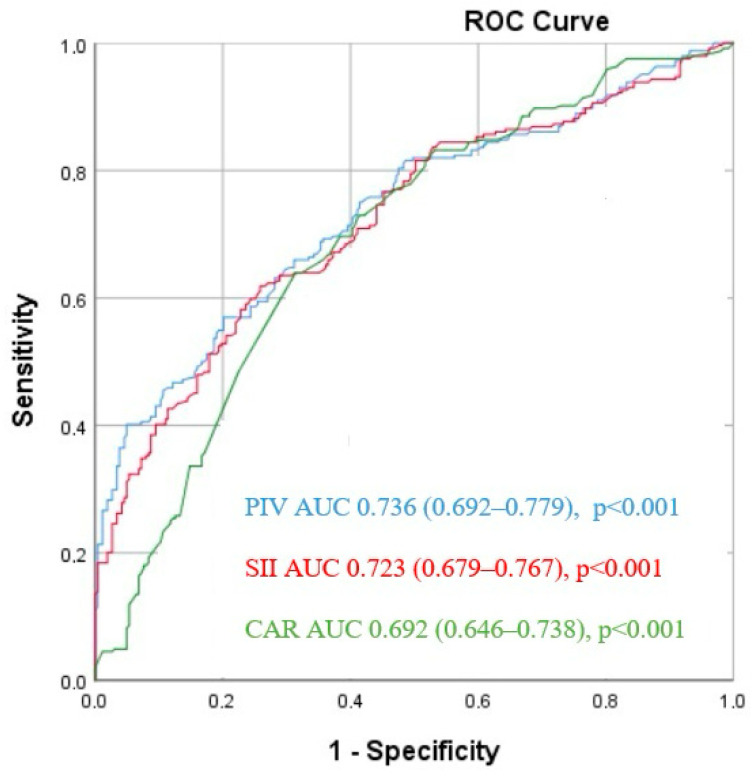
Receiver operating characteristic (ROC) curves for the pan-immune-inflammation value (PIV), systemic immune inflammation index (SII), and CRP/albumin ratio (CAR) for predicting saphenous vein graft stenosis.

The ROC analysis revealed that a PIV cut-off value > 315.5 predicted SVGS with 75.8% sensitivity and 68.6% specificity (area under the curve (AUC) = 0.736 [95% CI: 0.692–0.779], *p* < 0.001). Similarly, for SII, a cut-off value of 470.7 predicted SVGS with 73% sensitivity and 60.1% specificity (AUC = 0.723 [95% CI: 0.679–0.767], *p* < 0.001). In the case of CAR, a cut-off value of 0.95 demonstrated 70.9% sensitivity and 60.7% specificity in predicting SVGS among CABG patients (AUC = 0.692 [95% CI: 0.646–0.738], *p* < 0.001) (Figure 2).

## 4. Discussion

In this study, the frequency of DM was higher in patients with SVGS compared to those without, while inflammatory markers such as PIV, SII, CAR, and NLR were also found to be higher. Moreover, this study provides evidence that high PIV levels are at least as effective as the other markers in detecting SVGS and may even be slightly superior.

Appropriate indications and proper surgery significantly improve angina symptoms, quality of life, exercise capacity, and survival rates in patients with CAD undergoing CABG. In clinical practice, both arterial and venous conduits are frequently used in CABG surgery. The long-term benefit of CABG can be maximized with the use of arterial grafts, especially the left IMA [10,11]. However, since arterial grafts cannot always be used for all occluded vessels, SVGs remain an important alternative conduit. Nonetheless, the development of atherosclerosis in SVG is one of the most important problems responsible for SVG occlusion. This SVGF, which begins after one year, can be as high as 50% over a 10-year period [12]. Thrombosis in the first postoperative month, neointimal hyperplasia between one and twelve months, and increasing atherosclerosis after the twelfth month have been shown to be mechanisms responsible for SGVS [13,14,15].

The subsequent pathophysiological changes and the expression and secretion of pro-inflammatory cytokines induced by damage to the SVG wall due to predisposing factors for atherosclerosis (such as high blood pressure, diabetes, and obesity) support the formation of atheromatous plaques [16,17,18]. The role of inflammation in the initiation, progression, and complications of atherosclerosis is now an undeniable fact [19]. The relationship between systemic inflammation and arteriosclerosis has been demonstrated in various studies using different inflammatory markers.

PIV, a new inflammation parameter recently introduced, has been shown in studies to be a prognostic indicator of adverse outcomes in various types of cancer [20,21,22]. Publications investigating PIV, similar to inflammation markers such as NLR, CAR, and SII in patients with CAD, are increasingly being published [23,24,25,26,27]. There have also been studies on the association between PIV and other cardiovascular diseases. It has been reported to be a predictor of mortality in patients with hypertension and heart failure, as well as in predicting coronary slow flow [28,29,30,31]. Sari et al. [32] showed that it might be associated with the progression of aortic aneurysm in their study. At the same time, Yu et al. [33] claimed that it is a predictor of postoperative mortality in patients with acute type A aortic dissection. There are also studies suggesting that PIV can be used to distinguish between active/remission states in inflammatory diseases and/or to assess disease activity in some infectious/inflammatory diseases and that it can help assess mortality [34,35,36,37,38]. Neutrophils, monocytes, and platelets positively influence inflammation and the development of atherosclerotic plaques, while a decrease in lymphocytes has a negative effect.

PIV, which is associated with counts of neutrophils, monocytes, platelets, and lymphocytes, has been claimed by Liu et al. [23], in their study, to be predictive of adverse cardiovascular events in patients undergoing PCI following ST-elevation myocardial infarction (STEMI). Murat et al. [24] found it to be associated with long-term mortality following STEMI. Bayramoğlu et al. [25] found a relationship between PIV and no-reflow in patients undergoing PCI after STEMI. Cetinkaya et al. [26] demonstrated an independent relationship between PIV and the development of contrast-induced nephropathy (CIN) in patients with NSTEMI. Another recently published study showed that PIV can be used as an independent predictor of the development of coronary collateral circulation [27]. In our study, we also found that PIV is associated with SVGS in patients undergoing CABG and may be a slightly superior marker compared to other inflammatory markers. Our study is significant, as it is the first to examine this marker in this patient group.

Neutrophils have a significant impact on all processes related to atherosclerotic plaque. They may directly invade the plaque and also indirectly affect it by releasing proteolytic enzymes and arachidonic acid [39,40]. Endothelial damage triggers the participation of monocytes and lymphocytes, resulting in the development of fatty streaks consisting of lipid-loaded monocytes, macrophages (foam cells), and T lymphocytes [41]. Mononuclear cells (monocytes and lymphocytes) shift towards damage directly and/or together with cytokines, proteolytic enzymes and growth factors, and atherosclerotic plaque progresses [41]. Lymphopenia may arise from many processes, including reduced cell production caused by physiological stress, redistribution at the tissue level, or cell death [42]. As lymphocyte apoptosis rises inside the atherosclerotic plaque, the growth of the plaque advances and its ability to stabilize is compromised. Platelets modulate lymphocyte activation via intricate pathways and exert an effect on the functioning of different subsets of lymphocytes [43]. In addition, they facilitate the migration and proliferation of smooth muscle cells and monocytes by releasing granules that contain substances such as thrombin, cytokines, and growth factors [41,44,45]. PIV includes all three inflammatory parameters, including monocytes, which are considered the central component of local inflammation. Therefore, it creates a comprehensive inflammatory parameter that includes almost all blood cell types [46]. These findings suggest that PIV may potentially serve as a more accurate and comprehensive measure for predicting immunological and inflammatory/anti-inflammatory conditions in the individual.

Previous studies have shown that SII could be a more sensitive parameter in predicting the host’s immune and inflammatory status [23,47,48]. With the addition of monocytes, considered a core for local inflammation and known to affect the inflammation/anti-inflammatory cascade at every stage, a new inflammatory parameter incorporating almost all blood cell types has been obtained [46]. It is not unreasonable to think that SII, which consists of a single component of inflammatory markers (neutrophils or lymphocytes), two components of inflammatory markers (NLR or PLR), or a combination of three different inflammatory parameters, is a relatively poor prognostic predictor compared to PIV, which includes four different cells. Although we evaluated NLR, CAR, SII, and PIV in our study, some studies have evaluated different binary inflammatory markers and reported results supporting our hypothesis that PIV is a better marker [30,49,50,51]. PIV suggests that it could be a more sensitive and comprehensive parameter in predicting the host’s immune and inflammatory/anti-inflammatory status.

The results of the current study demonstrate that PIV, a useful, simple, easily measurable, and inexpensive indicator of the inflammatory state, is a strong inflammatory marker in predicting SVGS after CABG surgery. Larger and multicentric studies are required to better analyze all possible predictors of disease development.

This study has some limitations. Firstly, a relatively small number of patients were included, and it was a single-center, retrospective study. Secondly, PIV levels were calculated only at the time of hospitalization. Thirdly, potential atherosclerosis-influencing parameters such as VEGF, TGFα-β, and NO were not measured, lastly, there was no longer follow-up period.

## Figures and Tables

**Table 1:** Demographic characteristics of the study population.

	Saphenous Vein Graft Stenosis		
	Presence	Absence	*p*-Value *
Variables	244	263	
Age (years)	68.8 + 10.8	67.6 + 10.6	0.629
Gender, male (*n*, %)	178 (72.9%)	163 (62%)	0.833
Diabetes mellitus (*n*, %)	113 (46.3%)	96 (36.5%)	0.025
Hypertension (*n*, %)	170 (69.7%)	175 (66.5%)	0.450
Dyslipidemia (*n*, %)	61 (25%)	45 (17.1%)	0.197
Smoking (*n*, %)	92 (37.7%)	75 (28.5%)	0.310
Systolic blood pressure (mmHg)	124.5 + 14.4	122.8 + 14.3	0.186
Diastolic blood pressure (mmHg)	75.8 + 8.2	75.2 + 7.6	0.455
LVEF (%)	47.3 ± 10.5	48.9 ± 9.5	0.439
Postoperative medications			
Aspirin	219 (89.7%)	229 (87.1)	0.775
Β-blocker	206 (84.4%)	225 (85.5%)	0.695
Angiotensin–mineralocorticoid receptor antagonists	196 (78.6%)	213 (81%)	0.441
Statins	157 (64.3%)	166 (63.1%)	0.897
Clopidogrel	217 (88.9%)	225 (85.5%)	0.763

Values are expressed as *n* (%), median (interquartile range [IQR]), or mean ± standard deviation. *p* values were calculated using an independent samples *t*-test or the Mann–Whitney *U* test for continuous variables and a chi-squared test or Fisher’s exact test for categorical variables, as appropriate. LVEF, left ventricular ejection fraction. * *p*-value < 0.05 was considered statistically significant.

**Table 2:** Laboratory findings of the study population.

	Saphenous Vein Graft Stenosis		
	Presence	Absence	*p*-Value *
Number of patients	244	263	
Glucose (mg/dL)	158.1 + 31	147 + 40	0.065
Creatinine (mg/dL)	1.3 + 0.4	1.0 + 02	0.072
AST (U/L)	29.1 + 20	29.5 + 19	0.125
ALT (U/L)	26.7 + 12	28 + 117	0.204
Total cholesterol (mg/dL)	203 + 42	197 + 40	0.173
High-density lipoprotein cholesterol (mg/dL)	37.8 + 12	38.3 + 10	0.089
Low-density lipoprotein cholesterol (mg/dL)	121.8 + 37	116.8 + 33	0.071
Triglycerides (mg/dL)	161.5 + 89	155.1 + 84	0.389
Hemoglobin (mg/dL)	13.7 + 2	13.6 + 2	0.462
Platelets (10^3^/µL)	108 (84–152)	93 (75–116)	<0.001
WBC (10^3^/µL)	8.87 + 2.7	8.72 + 2.5	0.573
Monosit	0.66 (0.50–0.79)	0.65 (0.49–0.76)	0.460
PIV	525.2 (313.8–1110)	267 (177.7–447.6)	<0.001
C-reactive protein (CRP) (mg/L)	4.5 (2.3–5)	4.2 (2.6–5.7)	0.520
Albumin (g/L)	4.1 (3.5–4.2)	4.1 (3.7–4.3)	0.012
Neutrophil/lymphocyte ratio (NLR)	3 (1.9–5)	1.9 (1.4–2.7)	<0.001
CRP/albumin ratio (CAR)	1.1 (0.8–1.7)	0.7 (0.5–1.1)	<0.001
SII	713 (466–1229)	412 (305–626)	<0.001

Values are expressed as *n* (%), median (interquartile range [IQR]), or mean ± standard deviation. *p* values were calculated using an independent samples *t*-test or the Mann–Whitney U test for continuous variables and a chi-squared test or Fisher’s exact test for categorical variables, as appropriate. Abbreviations: ALT, alanine aminotransferase; AST, aspartate aminotransferase; WBC, white blood cell; PIV, pan-immune-inflammation; SII, systemic immune-inflammation index. * *p*-value < 0.05 was considered statistically significant.

**Table 3:** Angiographic data.

	Saphenous Vein Graft Stenosis		
	Presence	Absence	*p*-Value *
	244	263	
Distal anastomosis area			
Left anterior descending artery (*n*, %)	242 (99.1%)	261 (99.2%)	
Left circumflex coronary artery (*n*, %)	229 (93.8%)	241 (91.6%)	0.642
Right coronary artery	232 (95%)	247 (93.9%)	0.453
Left internal mammary artery usage (*n*, %)	242 (99.1%)	261 (99.2%)	0.882
Number of saphenous vein grafts	2.14 ± 0.61	2.06 ± 0.71	0.451
Average time after bypass (years)	8.4 + 1.6	7.2 + 1.8	<0.001

Values are expressed as *n* (%) and mean ± standard deviation. *p* values were calculated using independent samples *t*-test for continuous variables and a chi-squared test or Fisher’s exact test for categorical variables, as appropriate. * *p*-value < 0.05 was considered statistically significant.

**Table 4:** Univariate and multivariate logistic regression analysis to find independent predictors of saphenous vein graft disease.

Univariable Analysis Multivariable Analysis						
DM	1.052	1.052–2.141	0.025			
NLR ^a^	1.687	1.470–1.937	<0.001	1.726	1.485–2.006	<0.001
SII ^a^	1.002	1.002–1.003	<0.001	1.002	1.002–1.003	<0.001
Platelets ^a^	1.011	1.007–1.015	<0.001			
PIV ^a^	1.003	1.002–1.003	<0.001	1.003	1.002–1.003	<0.001
Albumin ^b^	0.534	0.325–0.877	0.013			
CAR ^b^	2.724	1.985–3.740	<0.001	2.948	2.060–4.217	<0.001
Average time after bypass				1.422	1.249–1.619	<0.001

Abbreviations: PIV, pan-immune-inflammation value; NLR, neutrophil/lymphocyte ratio; CAR, CRP/albumin ratio; SII, systemic immune-inflammation index. ^a,b^: These parameters are not entered into the model with each other to avoid multicollinearity.

## Data Availability

Data are contained within the article.
